# The cancer gene WWOX behaves as an inhibitor of SMAD3 transcriptional activity via direct binding

**DOI:** 10.1186/1471-2407-13-593

**Published:** 2013-12-11

**Authors:** Brent W Ferguson, Xinsheng Gao, Maciej J Zelazowski, Jaeho Lee, Collene R Jeter, Martin C Abba, C Marcelo Aldaz

**Affiliations:** 1 Department of Molecular Carcinogenesis, Science Park, The University of Texas M.D. Anderson Cancer Center, Smithville, TX 78957, USA; 2 CINIBA, Facultad de Medicina, Universidad Nacional de La Plata, La Plata, Argentina

**Keywords:** WWOX, SMAD3, Breast Cancer, TGFβ, ANGPTL4

## Abstract

**Background:**

The WW domain containing protein WWOX has been postulated to behave as a tumor suppressor in breast and other cancers. Expression of this protein is lost in over 70% of ER negative tumors. This prompted us to investigate the phenotypic and gene expression effects of loss of WWOX expression in breast cells.

**Methods:**

Gene expression microarrays and standard in vitro assays were performed on stably silenced WWOX (shRNA) normal breast cells. Bioinformatic analyses were used to identify gene networks and transcriptional regulators affected by WWOX silencing. Co-immunoprecipitations and GST-pulldowns were used to demonstrate a direct interaction between WWOX and SMAD3. Reporter assays, ChIP, confocal microscopy and *in silico* analyses were employed to determine the effect of WWOX silencing on TGFβ-signaling.

**Results:**

WWOX silencing affected cell proliferation, motility, attachment and deregulated expression of genes involved in cell cycle, motility and DNA damage. Interestingly, we detected an enrichment of targets activated by the SMAD3 transcription factor, including significant upregulation of *ANGPTL4*, *FST, PTHLH* and *SERPINE1* transcripts. Importantly, we demonstrate that the WWOX protein physically interacts with SMAD3 *via* WW domain 1. Furthermore, WWOX expression dramatically decreases SMAD3 occupancy at the *ANGPTL4* and *SERPINE1* promoters and significantly quenches activation of a TGFβ responsive reporter. Additionally, WWOX expression leads to redistribution of SMAD3 from the nuclear to the cytoplasmic compartment. Since the TGFβ target *ANGPTL4* plays a key role in lung metastasis development, we performed a meta-analysis of *ANGPTL4* expression relative to *WWOX* in microarray datasets from breast carcinomas. We observed a significant inverse correlation between *WWOX* and *ANGPTL4*. Furthermore, the *WWOX*^*lo*^*/ANGPTL4*^*hi*^ cluster of breast tumors is enriched in triple-negative and basal-like sub-types. Tumors with this gene expression signature could represent candidates for anti-TGFβ targeted therapies.

**Conclusions:**

We show for the first time that WWOX modulates SMAD3 signaling in breast cells *via* direct WW-domain mediated binding and potential cytoplasmic sequestration of SMAD3 protein. Since loss of WWOX expression increases with breast cancer progression and it behaves as an inhibitor of SMAD3 transcriptional activity these observations may help explain, at least in part, the paradoxical pro-tumorigenic effects of TGFβ signaling in advanced breast cancer.

## Background

*WWOX* (WW domain-containing oxidoreductase) was originally cloned by our laboratory because it was observed to reside in a chromosomal region (ch16q23) commonly affected by deletions in breast cancer [1]. Subsequently, it was concluded that the second most common chromosomal fragile site, FRA16D, spans the same locus as *WWOX*[1,2]. It was determined that FRA3B (*FHIT*) and FRA16D (*WWOX*) loci rank second and third respectively, only after the *CDKN2A* (*p16*) locus, as the chromosomal sites most commonly affected by hemi- and homozygous deletions in a genome wide study of over 740 cancer lines [3]. The high frequency of deletions affecting *WWOX* in multiple solid tumors is well documented [4-6]; additionally, translocations affecting *WWOX* are common in multiple myeloma [7]. Loss of WWOX expression is frequent in multiple tumor types including breast cancer. Importantly, it has been determined that over 70% of estrogen receptor alpha (ER) negative breast cancers express little or no WWOX protein, suggesting an inverse association between WWOX expression and increasing breast cancer aggressiveness [8,9].

WWOX behaves as a suppressor of tumor growth in some cancer lines [10-12]. Contradictory results were reported with *Wwox* KO mice that suffer from early life lethality; Aqeilan *et al.* reported osteosarcoma development in some *Wwox* KO newborn mice [13] whereas no neoplasias were detected in *Wwox* KO mice generated by our laboratory [14]. Furthermore, we recently demonstrated that no tumors develop spontaneously in mice targeted for conditional deletion of *Wwox* in the mammary gland [15]. Interestingly, *Wwox* ablation led to a significant inhibition of mammary gland ductal branching and impaired alveologenesis. Based on these studies, we concluded that *WWOX* does not behave as a classical tumor suppressor gene in the normal mammary gland. Therefore, in order to gain a better understanding of the role of WWOX in breast epithelium we investigated the cellular and molecular effects of modulating WWOX expression levels in normal, immortalized human breast cells.

## Methods

### Cell culture and reagents

All cell lines were obtained from the American Type Culture Collection (ATCC, Manassas, VA, USA) and validated by DNA fingerprinting. MCF10 cells (ATCC #CRL-10318) were cultured in DMEM/F12 supplemented with 5% fetal bovine serum, 100 μg/mL hydrocortisone, 10 μg/mL insulin, 20 ng/mL EGF, 1 ng/mL cholera toxin and 1% penicillin-streptomycin. MCF7 cells (ATCC #HTB-22) were cultured in modified IMEM supplemented with 10% fetal bovine serum. 184B5 cells (ATCC #CRL-8799) were cultured in MEBM. Recombinant human TGFβ1 was purchased from R&D Systems.

### shRNA-mediated WWOX silencing in MCF10 cells

Cells were infected with the following shRNA-expressing GIPZ lentiviruses (Open Biosystems) at an MOI of 5: scrambled control shRNA (RHS4348), sh*WWOX*-A (V2LHS_255213); sh*WWOX*-B (V2LHS_255229) or sh*WWOX* (V2LHS_255213 and V2LHS_255229). Cells were infected according to manufacturer’s instructions. Stably *WWOX* silenced cells and controls were selected with 2 μg/ml puromycin and WWOX protein level was assayed by western blot.

### Doxycycline-inducible WWOX expression system and other transient transfections

pLVX-Tight-Puro from Clontech’s Tet-on advance system (Clontech, Mountain View, CA) was used to construct inducible WWOX expression. Full-length human *WWOX* cDNA was amplified and inserted using BamH1/EcoR1 restriction enzyme sites. Lentiviral stocks were made according to manufacturer’s protocol. MCF10 cells were either stably or transiently infected by the lentiviruses carrying the target cassettes and subjected to selection with 2 μg/ml puromycin. One μg/ml of doxycycline were used to induce WWOX expression.

Transient transfections were performed using FuGene 6 transfection reagent (Promega) and plasmids used were: pCMV5b-FLAG-*SMAD3* (Addgene plasmid 11742) [16], 3TP-LUX (Addgene plasmid 11767) [17], pRL Renilla luciferase and pcDNA-Myc-*WWOX*.

### Microarray data processing, bioinformatics and statistical analyses

Total RNA was extracted from 3 biological replicates each of MCF10 scrambled (Scr), MCF10 shWWOX-A and MCF10 shWWOX-B using the RNeasy Mini kit (Qiagen). Briefly, 2 μg of RNA from each of WWOX–silenced sublines labeled with Cy5 were individually hybridized on Agilent Whole Human Genome 4X44K microarrays to analyze ~40000 transcripts (Agilent Technologies, Palo Alto, CA, USA) using the RNA derived from the corresponding MCF10 Scr sample (labeled with Cy3) as reference. For RNA labeling, we used the Quick Amp Kit (Agilent Technologies, Palo Alto, CA) by following the manufacturer’s protocol. The hybridization steps were carried out according to the Agilent protocol and images were scanned using a Genepix 4000B microarray scanner (Axon Instruments, Foster City, CA, USA). Image analysis and initial quality control were performed using Agilent Feature Extraction Software v10.2. Raw datasets have been submitted to NCBI GEO database with accession number GSE47371. We used the limma Bioconductor package for background adjustment (normexp method), within (Loess algorithm) and between (quantiles method) arrays normalization [18]. To identify significantly up- or down-modulated genes within the hybridized samples (MCF10 shWWOX-A vs. Scr and MCF10 shWWOX-B vs. Scr) we employed the one-class Rank Products' test (q-value < 0.05; Fold change > 2) [19]. Statistical analyses were done with the MultiExperiment Viewer software (MeV 4.8) [20]. Differentially expressed genes derived from both analyses were compiled into one Excel spreadsheet pivot Table for comparison of overlapping data between MCF10 shWWOX-A and MCF10 shWWOX-B WWOX sub-lines. The number and identity of genes commonly affected in both models was determined. We used the normal approximation to the binomial distribution as previously described [21] to calculate whether the number of matching genes derived from each pairwise comparison was of statistical significance (q < 0.05). Datasets were then uploaded to IPA software for automated functional annotation and gene enrichment analysis [22]. In addition, we employed Enrichr online resource [23] for ChIP enrichment analysis [24].

### Clonal growth, attachment and cell motility assays

For clonal growth assays, 500 cells were plated into individual wells of a 6-well plate. After 9 days of culture, colonies were fixed and stained with crystal violet. Digital images were used to determine the number and area of growing colonies using ImageJ software 1.46 (NIH).

For attachment assays, cells (4×10^4^ per well) were seeded in serum-free medium on fibronectin, collagen IV or laminin-coated 96-well plates (BD-BioCoat; BD) and incubated for 120 min at 37°C/5% CO_2_. Adherent cells were fixed at different time-points by adding a cold 10% TCA solution and then processed according to the sulforhodamine B (SRB) assay (Sigma-Aldrich).

To assess cell motility we conducted a standard wound-healing assay. Briefly, 1×10^6^ cells were seeded in each well. After cells adhered the FBS concentration in the medium was reduced to 2% to decrease cell proliferation. Two scratch wounds were made in each well. Images of the same fields were collected at 0 and 24 hrs. Wound area expressed as percent of field of view was quantified using the ImageJ software.

### Real-time Q-PCR, ELISA, Western blotting and antibodies

RNA isolation and Real-time PCR was performed as previously described [15]. Real-time assays were performed using Sybr Green and the following primer sets: FST F 5′-GCCACCTGAGAAAGGCTACC-3′, FST R 5′-TTACTGTCAGGGCACAGCTC-3′, ANGPTL4 F 5′- CACAGCCTGCAGACACAACT -3′, ANGPTL4 R 5′- AAACTGGCTTTGCAGATGCT -3′, PTHLH F 5′-CGCTCTGCCTGGTTAGACTC-3′, PTHLH R 5′-AGAATCCTGCAATATGTCCTTGG-3′, SERPINE1 F 5′-GACCGCAACGTGGTTTTCTC-3′, SERPINE1 R 5′-CATCCTTGTTCCATGGCCCC-3′, 18S rRNA F 5′-ACGGAAGGGCACCACCAGG-3′ and 18S rRNA R 5′-CACCAACTAAGAACGGCCATGC-3′. Experiments were done in triplicate and normalized to 18S rRNA expression.

Levels of FST and ANGPTL4 proteins in conditioned medium were determined using the FST Quantikine ELISA kit and the ANGPTL4 DuoSet ELISA kit (R&D Systems) according to manufacturer’s protocols. Briefly, 4×10^5^ cells were seeded in phenol red-free DMEM/F12 medium supplemented with charcoal-stripped serum (5%) and adequate growth factors under normal conditions for 72 hrs before collection of conditioned medium.

Western blotting was performed under standard conditions by loading 20 μg of total protein per lane and transferring to PVDF membranes. Primary antibodies used were: rabbit anti-WWOX (Aldaz lab), rabbit anti-SMAD3 (Cell Signaling), mouse anti-actin (Sigma-Aldrich) and mouse anti-Myc (Origene). Secondary antibodies used were: anti-rabbit HRP (Jackson Labs) anti-mouse HRP (K&P Labs), anti-rabbit Alexa 594 and anti-mouse Alexa 488 (Invitrogen).

### Co-immunoprecipitation, GST-pulldowns and Luciferase assays

For co-immunoprecipitation, cells were lysed with a buffer containing 50 nM Tris–HCl pH 7.4, 100 mM NaF, 10 mM EDTA, 10 mM Na_3_VO_4_, 2 mM PMSF, 1% NP-40 and 0.5% TritonX-100. Immunoprecipitations were carried out with Protein A/G beads and washed five times in the same buffer. Construction and purification of GST fusion proteins was performed as previously described [25]. Pull-down assays were performed using immobilized purified GST or GST fusion proteins incubated with total cell lysate from MCF10 cells transfected with 1 μg of pCMV5b-Flag-SMAD3 plasmid for 48 hours.

For luciferase assays, MCF10 cells stably infected with the described Dox-inducible WWOX expression system were exposed to 1 μg/mL doxycycline for two days (or no treatment). Cells were then co-transfected with 3TP-LUX and pRL Renilla luciferase expressing control vector. Serum-free media was applied and cells were then exposed to 10 ng/mL TGFβ1 (or vehicle treatment) for 8 hours. Luciferase assays were performed according to Dual-Luciferase Assay protocol (Promega).

### Chromatin immunoprecipitation (ChIP)

MCF10 cells transiently infected with the described Dox-inducible WWOX expression system were exposed to 1 μg/mL Dox for one day (or no treatment), changed to serum-free media for 16 hours then exposed to 10 ng/mL TGFβ1 for 4 hours (or vehicle treatment). ChIP was performed as described elsewhere [26].

Real-time PCR was performed to assay SMAD3 occupation at promoter elements via the percent input method. Primers used for ChIP qPCR for the region 2000 bases upstream from the *ANGPTL4* transcriptional start site were: F: 5′-GATTTGCTGTCCTGGCATCT-3′ and R: 5′-CTCCAAGCCAGCTCATTCTC-3′. Primers for the SMAD binding element of the *SERPINE1* promoter were: F: 5′-GGGAGTCAGCCGTGTATCAT-3′ and R: 5′-TAGGTTTTGTCTGTCTAGGACTTGG-3′ [27].

### Confocal microscopy

Cells transiently transfected with pcDNA-Myc-*WWOX* were seeded on round, glass coverslips in 12-well plates, serum starved for 12 hours, treated with 20 ng/μL TGFβ1 for 1 hour, fixed for 15 min in 4% PBS-buffered paraformaldehyde, permeabilized with 0.05% Triton X-100 in PBS (PBS-T) for 5 min, blocked with 1% bovine serum albumin (BSA), and incubated with rabbit anti-SMAD3 (Cell Signaling) overnight at 4°C then mouse anti-Myc (Origene) for one hour at room temperature. AlexaFluor-conjugated secondary antibodies were applied for 2 hours at room temperature. Cells were washed three times in PBS-T, DAPI solution applied, washed three more times then mounted in Prolong Gold Anti-Fade (Invitrogen) on a microscope slide. Confocal microscopy was done on a Zeiss LSM510 META confocal microscope with 100X plan-apochromatic objective and oil immersion. Images were acquired in sequential mode and single-color controls were used to verify absence of crosstalk and bleed-through.

### *WWOX* and *ANGPTL4* expression meta-analysis in breast cancer datasets

To perform a comparative analysis of *WWOX* and *ANGPTL4* expression in breast cancer, we analyzed 819 primary carcinomas obtained from three independent studies available in public databases. The fRMA preprocessed expression matrixes of the studies GSE26639 (n = 226), GSE21653 (n = 266), and GSE20685 (n = 327) were downloaded from the InSilico database [28]. These gene expression profiles were obtained using the Affymetrix HG U133 Plus2 platform (GPL570). *WWOX* and *ANGPTL4* mRNA expression levels were estimated by using the mean expression values of the Affymetrix probes for each gene. We employed the Gaussian Mixture Model to identify bimodal distributions in the expression levels of both genes [29]. Heatmap visualization of *WWOX* and *ANGPTL4* expression profiles was done with the MultiExperiment Viewer software (MeV 4.8).

## Results

### WWOX silencing in breast cells affects clonal growth, adhesion and motility

**Figure 1:**
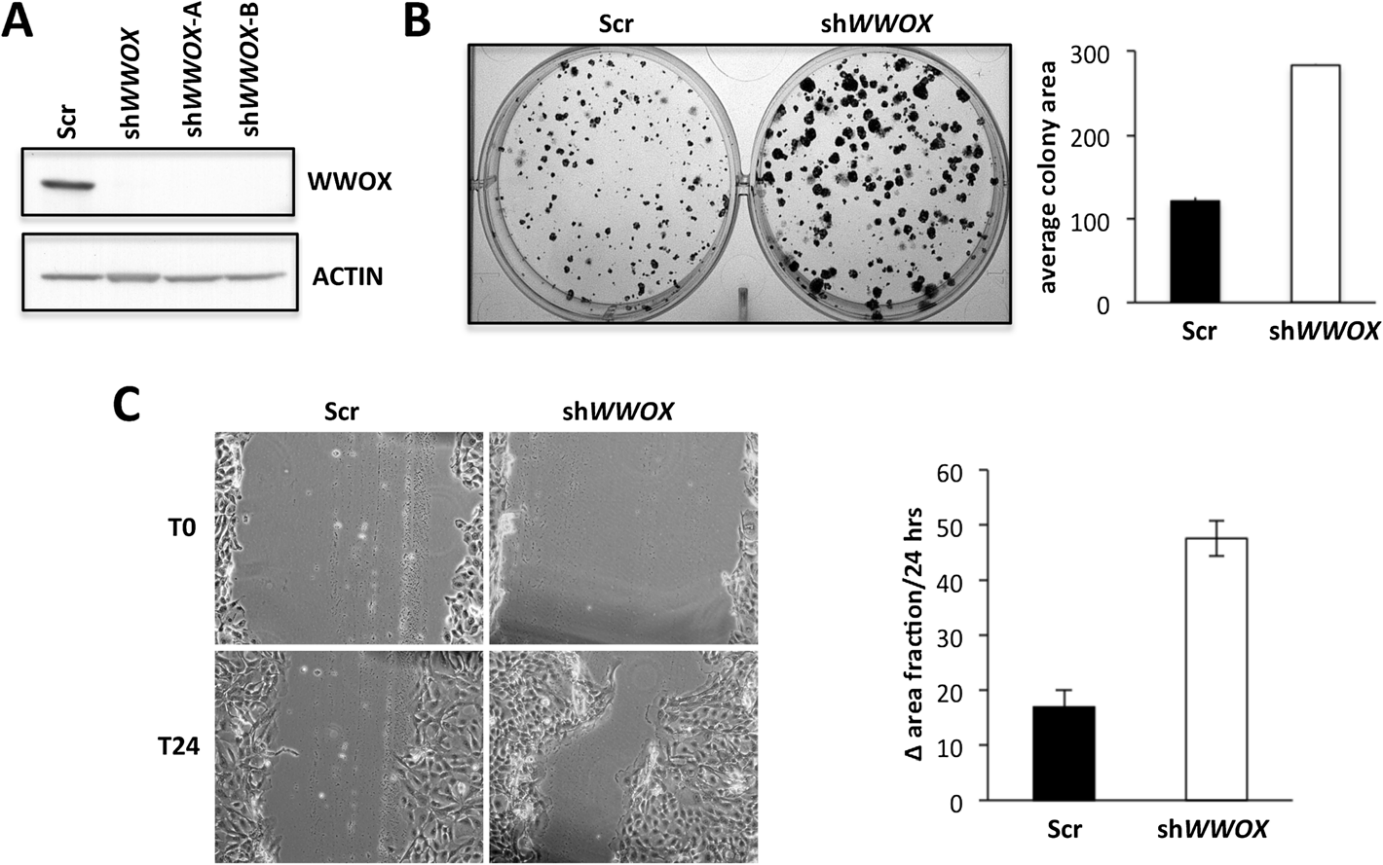
**Silencing of WWOX results in increased clonal growth, decreased attachment and increased cell motility. (A)** Western blot demonstrating WWOX silencing in cell extracts from MCF10 control (Scr) and three independent stably WWOX-silenced cell lines. **(B)** Effect of WWOX-silencing on clonal growth. Five hundred cells were seeded in each well of a 6-well plate for each subline and allowed to grow for 9 days. Representative image of both conditions is shown. Bar graph represents the average of three independent experiments +/- SEM. **(C)** Scratch wound healing assay. Results of four separate experiments done in biological triplicates (two wounds per well). Depicted is the average percent of the difference between T0 and T24 from all experiments (±SEM).

In order to gain insight into the consequences of loss of WWOX expression we investigated the effects of WWOX silencing in normal breast epithelial cells. To this end, we used an shRNA-mediated approach to stably knockdown expression of WWOX in the normal human breast cell line MCF10. Three independent stable *WWOX* shRNA-expressing cell lines were generated (sh*WWOX*, sh*WWOX*-A and sh*WWOX*-B) and one scrambled shRNA control. All three stably *WWOX*-silenced cell lines showed a decrease of 80-90% WWOX protein expression levels (Figure 1A).

We first investigated the effects of *WWOX* silencing on the clonal growth of the MCF10 cells. We did not detect differences in clonogenicity (i.e. number of colonies) but found that MCF10 *WWOX*-silenced cells proliferate more rapidly forming larger colonies than their control scrambled shRNA counterparts (Figure 1B). *WWOX*-silenced cells also displayed decreased attachment to extracellular matrix components such as laminin, collagen IV and fibronectin (Additional file 1) and were significantly more motile, repopulating the wound faster in the scratch wound-healing assay when compared with controls (Figure 1C). In summary, our data suggests that WWOX ablation influences cell proliferation, adhesion and motility of breast cells.

### Gene expression changes in normal human breast cells silenced for WWOX expression

**Figure 2:**
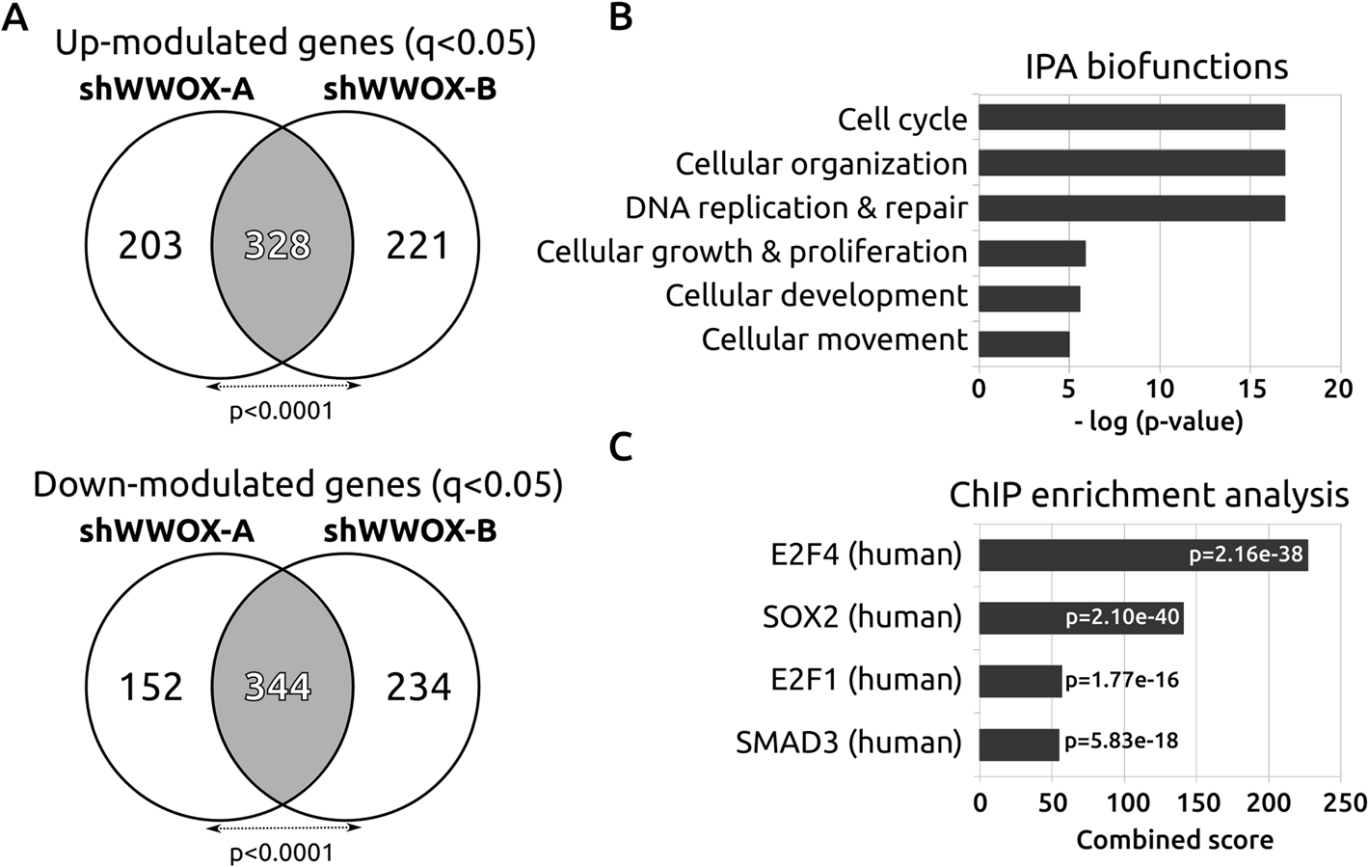
**Effects of WWOX silencing on gene expression. (A)** Venn diagram showing the overlap between transcripts up- or down-modulated in two independent WWOX-silenced MCF10 sublines (fold-change >2.0, q < 0.05). **(B)** Top predicted biofunctions deregulated in WWOX-silenced cells. Bar graph represents –log(p-value) for each biofunction. Biofunction prediction from IPA software. **(C)** ChIP enrichment analysis (ChEA) from the commonly deregulated gene list. Bars represent the four transcription factors with the highest combined scores calculated by the Enrichr resource, i.e. transcription factors more likely associated with the majority of gene expression changes observed (see also Additional file 2).

To determine global gene expression changes as a result of *WWOX* silencing in normal human breast cells we performed microarray studies. We compared two independent shRNAs (sh*WWOX*-A and sh*WWOX*-B) targeting different regions of the *WWOX* transcript as a means of ruling out any potential off-target effects. The statistical analysis of the sh*WWOX*-A and sh*WWOX*-B gene expression profiles identified 328 commonly up-modulated and 344 commonly down-modulated genes (q value < 0.05) in the two *WWOX* stably silenced cell lines (Figure 2A) (Additional file 2). We used the Ingenuity Pathway Analysis (IPA) resource for automated annotation and classification of the common differentially expressed genes. Among the statistically significant top biofunctions deregulated in *WWOX*-silenced cells, we identified cell cycle/proliferation, DNA replication, recombination and repair as well as cellular movement (Figure 2B). These biofunctions were consistent with the results from our phenotypic assays as markers of proliferation such as M*KI67* and *PCNA* were both significantly upregulated in WWOX silenced cells (Additional file 2). To identify affected transcriptional regulatory networks, we performed a ChIP enrichment analysis (ChEA) from the commonly deregulated gene list. Briefly, ChEA identifies over-representation of transcription factor targets from a mammalian ChIP-X database [24]. ChEA allowed us to identify a set of transcription factors that are the most likely to have regulated WWOX associated gene expression changes. We detected a statistically significant enrichment of E2F family members, SOX2 and SMAD3 gene targets (Figure 2C) (Additional file 2).

### Upregulation of SMAD3 target genes in *WWOX* silenced cells

**Figure 3:**
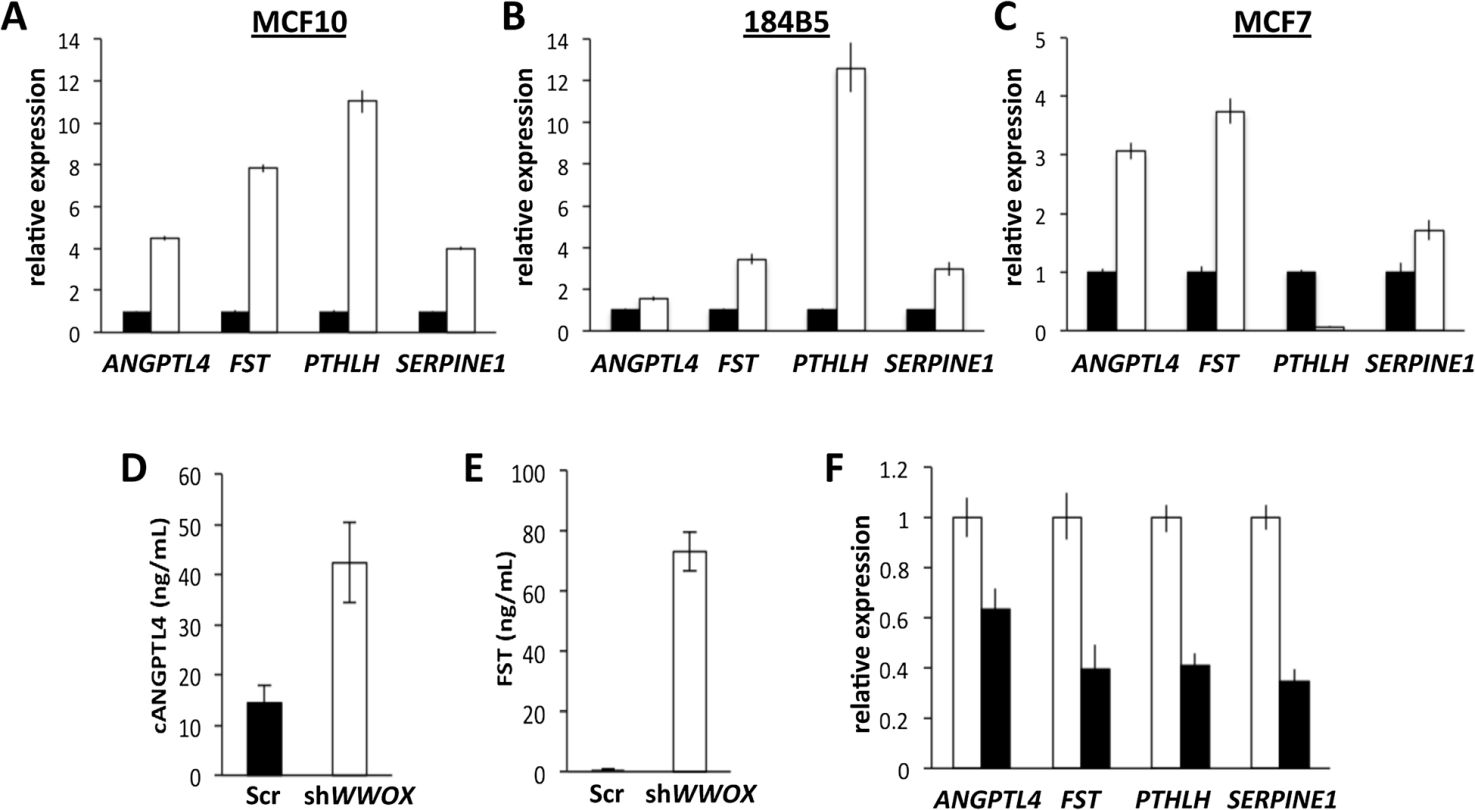
**WWOX silencing results in increased expression of SMAD3 target genes. (A-C)** Validation of increased gene expression of SMAD3-regulated genes in MCF10 **(A)** shWWOX subline (white bars) compared to Scr shRNA control (black bars) by Real Time qPCR. mRNA from three biological replicates of each stable cell line were used for quantitation, 18S rRNA was used as normalization control, (p < 0.01 for all genes). Further real-time PCR validation of SMAD3 target upregulation in WWOX-silenced (shWWOX-B, white bars) or Scr shRNA control (black bars) 184B5 normal breast cells **(B)** or MCF7 breast cancer cells **(C)**. Significant upregulation was seen for all target genes in all WWOX-silenced samples with the exception of *PTHLH* expression in MCF7 cells. **(D)** ELISA assay for quantitation of ANGPTL4 protein concentration in culture media from each MCF10 subline after 72 hours in culture (p < 0.01). **(E)** ELISA assay for FST protein concentration in culture media from MCF10 sublines. **(F)** Reversion of SMAD3 target gene upregulation by inducible ectopic WWOX expression in MCF10 shWWOX-A cells. Real-Time PCR analysis of MCF10 shWWOX-A cells transiently transduced with a DOX-inducible WWOX expression system. With induction of ectopic WWOX expression (black bars) or without WWOX expression (white bars). 18S rRNA used as normalization control (p < 0.05 for all genes).

Interestingly, of the top 25 most upregulated genes in *WWOX*-silenced cells 40% were SMAD3 target genes (Additional file 2). Thus, SMAD3 appears as one of the top transcriptional regulators likely responsible for many of the gene expression changes detected by our microarray analysis. Among the group of most significantly upregulated SMAD3 target genes we identified: *FST* (5.2 fold), *PTHLH* (3.6 fold), *ANGPTL4* (3.5 fold) and *SERPINE1* (2.5 fold). Real Time RT-PCR validations are shown in Figure 3A. In order to explore whether this finding was exclusive of MCF10 cells, we stably silenced *WWOX* expression in another normal breast epithelial cell line (184B5) and a breast cancer line (MCF7). Interestingly, we observed a similar SMAD3 target gene upregulation induced by *WWOX* silencing in those two breast derived cell lines as well (Figure 3B-C).

Since the four aforementioned SMAD3 target genes all produce secreted proteins, we tested by ELISA the production of two of these proteins (ANGPTL4 and FST) and detected significant increased secretion of these proteins in cultured media from *WWOX* silenced cells (Figure 3D-E).

To further investigate whether transcription of these genes is regulated by WWOX expression status we transiently transduced MCF10 *WWOX*-silenced cells with a lentiviral, *WWOX* doxycycline-inducible system. We determined that mRNA levels of each of the four genes assayed decrease significantly when WWOX protein is re-expressed (Figure 3F). Overall we demonstrate that WWOX expression status influences the expression of subsets of SMAD3-regulated genes.

### WWOX inhibits TGFβ induced transcriptional activation and decreases SMAD3 promoter occupancy

**Figure 4:**
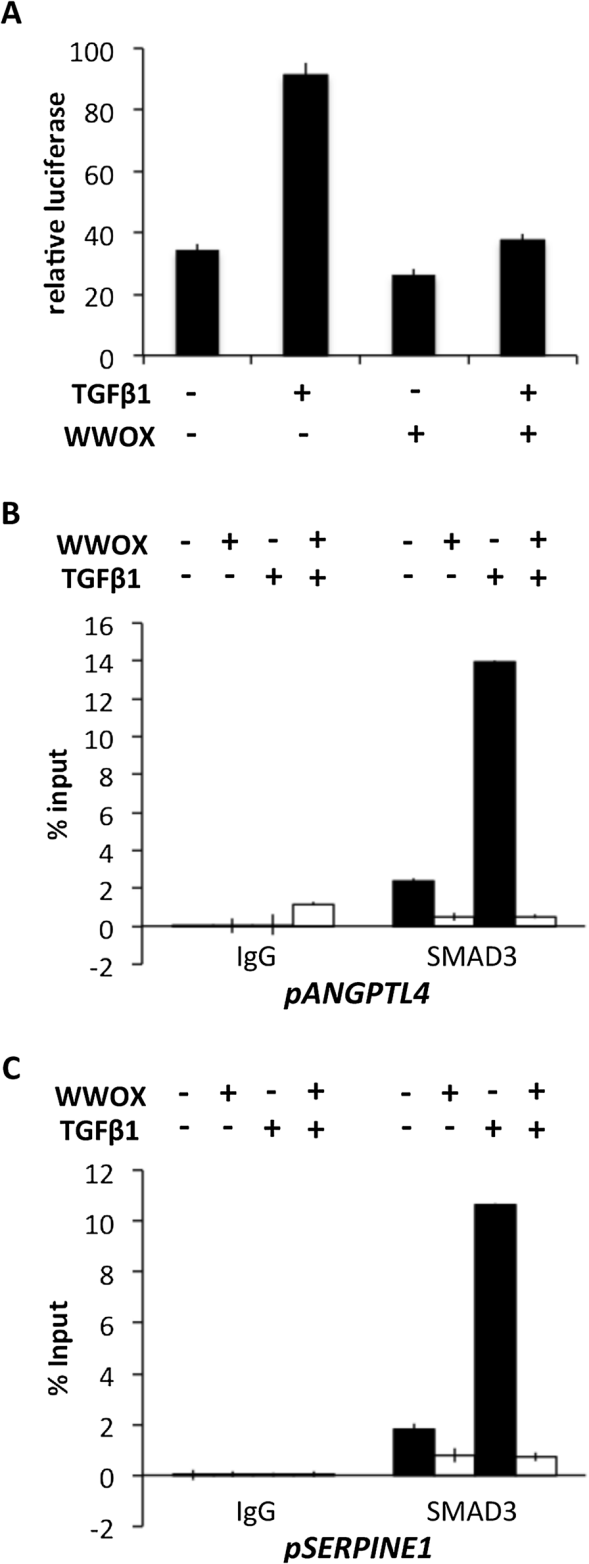
**WWOX inhibits TGFβ-dependent transcription and decreases SMAD3 occupancy at target gene promoters. (A)** 3TP-LUX luciferase reporter assay in MCF10 cells treated with or without doxycycline to induce WWOX expression and with or without TGFβ1 for 8 hours as indicated. Experiment done in triplicate. **(B-C)** Chromatin immunoprecipitation of SMAD3 (or IgG control) followed by qPCR in MCF10 cells transiently infected with the doxycycline-inducible WWOX expression lentivirus system. Primers used for qPCR span SMAD binding elements in the *ANGPTL4* promoter **(B)** or the *SERPINE1* promoter **(C)**. Error bars represent SD for all graphs.

Since SMAD3 is a known TGFβ activated transcription factor we investigated whether WWOX affects TGFβ-dependent transcription using the 3TP-LUX luciferase reporter. This plasmid contains a strong TGFβ-responsive element from the *SERPINE1* (also known as Plasminogen Activator inhibitor, PAI) promoter and is routinely used to assay TGFβ signaling [17]. Indeed, we found that dox-inducible expression of WWOX protein in MCF10 cells significantly quenched TGFβ-dependent luciferase expression (Figure 4A).

We then asked whether WWOX expression in MCF10 cells would affect binding of SMAD3 to known DNA responsive elements on the *ANGPTL4* and *SERPINE1* promoters [27]. Using chromatin immunoprecipitation (ChIP) we observed, as expected, a significant increase in SMAD3 presence at both promoters upon TGFβ1 treatment. However, when WWOX expression was induced we found a dramatic loss of SMAD3 occupancy at both promoters (Figure 4B-C). These results demonstrate that WWOX protein expression affects SMAD3 protein availability for binding effector promoter elements both in the idle state and upon TGFβ1 stimulation.

### WWOX interacts with SMAD3 via WW domain 1

**Figure 5:**
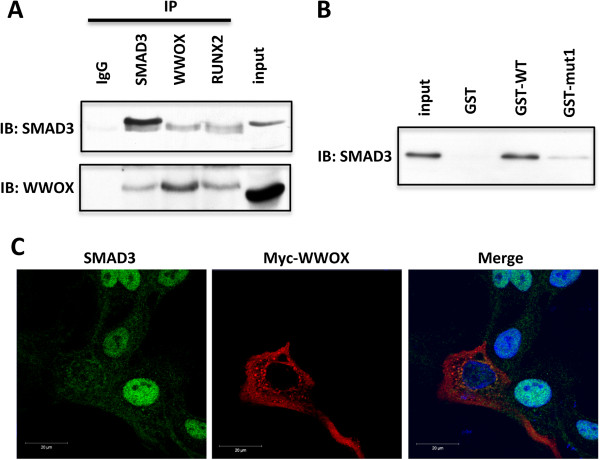
**WWOX binds to SMAD3 and relocalizes it to the cytoplasm. (A)** Co-immunoprecipitation of endogenous WWOX and SMAD3 from MCF10 cells. Whole cell lysates were immunoprecipitated with either rabbit IgG (control), anti-WWOX, anti-SMAD3 or anti-RUNX2 antibodies. The immunoprecipitates were immunoblotted with anti-WWOX and anti-SMAD3 antibodies as indicated. RUNX2 co-IP was used as a positive control for both WWOX and SMAD3 interactions. **(B)** MCF10 cells were transfected with Flag-SMAD3 and pulldowns were performed with the indicated GST fusion proteins either wild-type WW1 and 2 domains (GST-WT) or mutant WW1 (W44F/P47A) (GST-mut1) or GST alone. Bound protein was detected by SMAD3 immunoblot. **(C)** Confocal microscopy of MCF10 cells transfected with pcDNA-Myc-*WWOX* and treated with TGFβ1. Note how SMAD3 (green) localizes to the nucleus in cells with no Myc-WWOX expression (red) but undergoes intracellular redistribution mostly to the cytoplasmic compartment in cells with ectopic WWOX expression (red). Images taken using a Zeiss LSM510 META confocal microscope and the 100X objective.

The first WW domain of WWOX is a Class I WW domain known to bind to PPXY motifs on target proteins in a phosphorylation-independent manner [25,30]. Since the SMAD3 protein contains a ^181^PPGY^184^ motif we investigated whether WWOX and SMAD3 proteins physically interact. Indeed co-immunoprecipitation of endogenous WWOX and SMAD3 proteins from MCF10 cell extracts demonstrates a strong interaction between the two proteins (Figure 5A). The SMAD3 coactivator RUNX2 is known to bind both SMAD3 and WWOX [31,32] thus it was used as a positive control for both co-immunoprecipitations. To determine whether the observed interaction is dependent upon WW1 domain of WWOX, GST-pulldown experiments were performed. We observed that SMAD3 from MCF10 whole cell lysates readily binds to the wild type WW domains of WWOX but the interaction is lost when the first WW domain is mutated (W44F/P47A) (Figure 5B).

### WWOX expression induces intracellular SMAD3 redistribution

WWOX is a cytoplasmic protein [10,25] while SMAD3 is predominantly found in the nuclear compartment. To determine whether WWOX affects SMAD3 protein subcellular localization, we used confocal microscopy to analyze SMAD3 intracellular distribution with or without WWOX ectopic expression. As expected, in MCF10 cells treated with TGFβ1, we found a predominantly nuclear staining for SMAD3 (Figure 5C). Interestingly however, induction of WWOX expression led to a cellular redistribution of SMAD3 protein levels shifting from the nuclear to the cytoplasmic compartment and perinuclear colocalization with WWOX.

### *WWOX* and *ANGPTL4* are inversely correlated in breast cancer and the *Wwox*^*lo*^*/ANGPTL4*^*hi*^ cluster is enriched in TNBC and basal-like cancers

**Figure 6:**
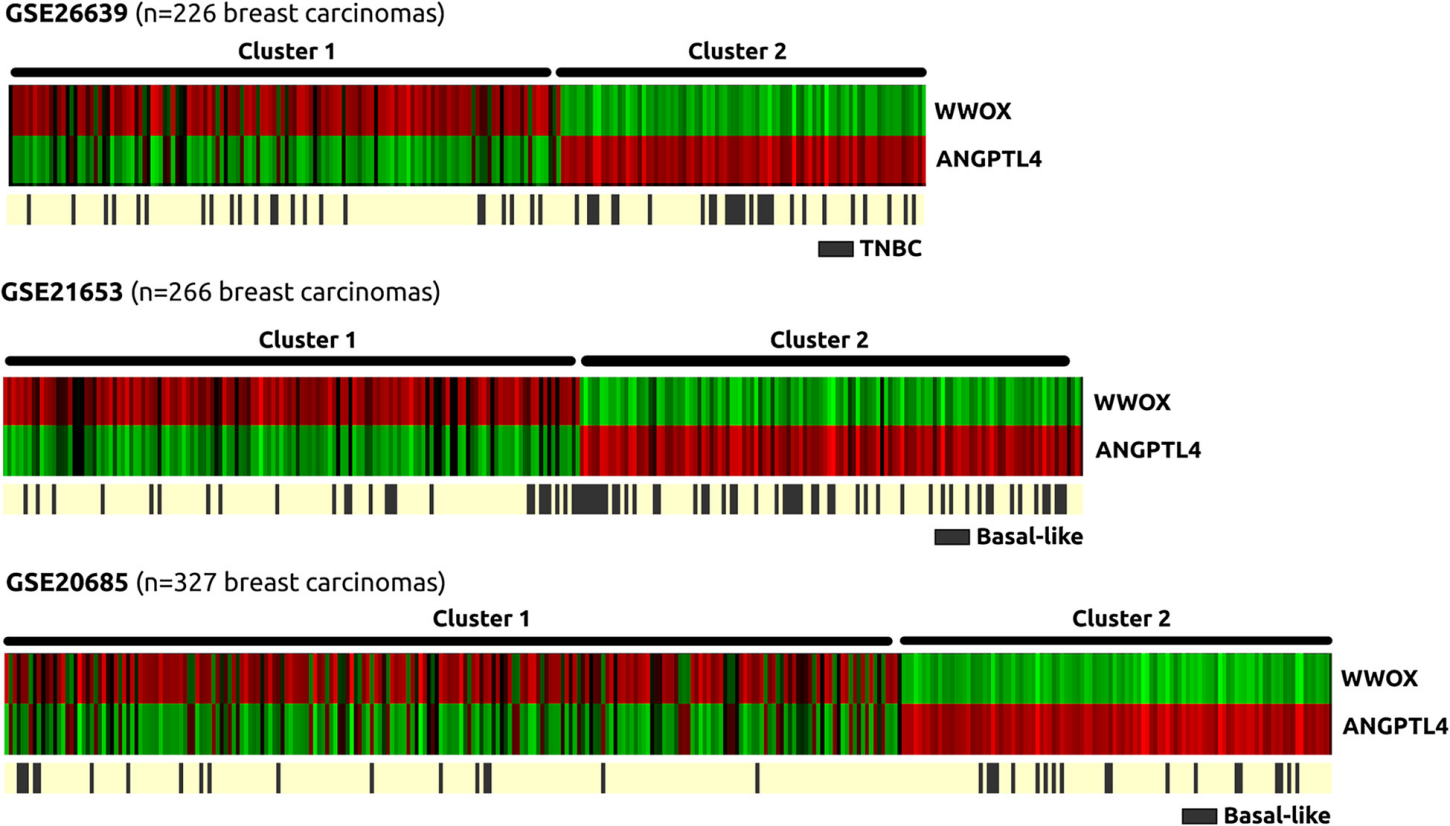
***WWOX *AND *ANGPTL4 *expression meta-analysis in breast cancer datasets.** Comparative analysis of *WWOX* and *ANGPTL4* expression in three independent gene expression studies of primary breast carcinomas. Unsupervised clustering resulted in two main groups, Cluster 1: *WWOX*^*hi*^*/ANGPTL4*^*lo*^ and Cluster 2 *WWOX*^*lo*^*/ANGPTL4*^*hi*^ according to their gene expression profiles. We identified a statistically significant enrichment of TNBC and basal-like breast carcinomas in cluster 2 from each dataset (p < 0.05).

Given the relevance of ANGPTL4 as a key determinant of lung metastatic phenotypes for breast cancer cells [33,34] and our observations of a clear inverse behavior between WWOX and ANGPTL4 at the transcript and protein level, we investigated whether this inverse relationship extended to breast cancers. To this end we performed a meta-analysis using three independent gene expression breast cancer datasets representing a total of 819 breast carcinoma samples. Unsupervised clustering of these samples showed the emergence of two defined clusters, cluster 1: *WWOX*^*hi*^*/ANGPTL4*^*lo*^ and cluster 2: *WWOX*^*lo*^*/ANGPTL4*^*hi*^ representative of a statistically significant negative correlation between *WWOX* and *ANGPTL4* expression (Figure 6). Further analysis of breast tumor subtypes determined that the *WWOX*^*lo*^*/ANGPTL4*^*hi*^ cluster demonstrates a significant enrichment of triple-negative breast cancer (TNBC) and basal-like tumors (Cluster 2, p < 0.05). Overall, our analysis reveals a significant inverse correlation between *WWOX* and *ANGPTL4* transcript levels in breast cancer patient samples and that tumors with the *WWOX*^*lo*^*/ANGPTL4*^*hi*^ signature correlate with breast cancer subtypes characterized by poor prognosis.

## Discussion

It is clear that expression of WWOX is lost in breast cancer and that this loss becomes more frequent as the disease progresses [8,9,35,36]. Thus, we feel it is important to understand the functions of WWOX in normal breast cells and the effects of loss of expression of this protein in breast cancer progression. In this study, we have described the multiple consequences of *WWOX* silencing in normal human breast cells. *WWOX* knockdown leads to a pro-transformation phenotype with increased proliferation, decreased attachment to ECM substrates and increased cell motility. These phenotypes were supported by corresponding changes in gene expression as genes involved in cell cycle, DNA damage response and cell motility were found deregulated in WWOX silenced cells.

ChIP enrichment analysis identified SMAD3 as one of the most over-represented transcription factors responsible for many of the observed gene expression changes. Well known SMAD3 target genes such as *FST, ANGPTL4, PTHLH* and *SERPINE1* were found significantly upregulated upon WWOX silencing. Interestingly, *ANGPTL4*, *PTHLH* and *SERPINE1* have all been shown to be involved in breast cancer progression and metastasis [33,37,38]. We observed that these specific gene expression changes detected in WWOX knockdown cells can be reverted upon WWOX re-expression. Furthermore, we showed that WWOX protein expression significantly decreases SMAD3 promoter occupancy at target DNA elements and significantly decreases the response of a TGFβ luciferase reporter.

These observations lead us to investigate whether WWOX and SMAD3 physically interact with each other. Indeed, we demonstrate for the first time that WWOX is able to bind SMAD3 via the first WW domain and likely modulates SMAD3 transcriptional activity by cytoplasmic sequestration.

The effect of TGFβ signaling in breast cells has been described as paradoxical since it acts as an inhibitor of growth in normal mammary epithelium [39] but transitions to being an enhancer of tumor progression in advanced breast cancer stages [40-42]. The mechanisms behind this dichotomous behavior are poorly understood [43]. In normal mammary epithelial cells TGFβ inhibits cell growth by inducing the expression of cell cycle inhibitors such as *CDKN2B* (*p15*) and *CDKN1A* (*p21*) and repressing the expression of cell cycle activators such as *MYC*[44-46]. On the other hand, in advanced-stage breast cancer the growth inhibitory effects of genes such a p15 and p21 are no longer effective and different subsets of pro-oncogenic and pro-metastatic genes are activated by TGFβ [40-42]. In fact the majority of breast cancers demonstrate active signaling through the TGFβ pathway and some tumors secret high levels of TGFβ [40].

SMAD protein family members are known to be regulated by a number of WW-domain containing proteins such as YAP, PIN1, NEDD4L and SMURF1/2 [47,48]. YAP and PIN1 interact with SMADs in a phosphorylation-dependent manner and stabilize SMAD-cofactor binding at promoter elements to enhance transcriptional effects [47]. NEDD4L and SMURF1/2 are E3 ubiquitin ligase proteins responsible for SMAD protein turnover [43,47]. WWOX, also a WW domain containing cytoplasmic protein, is known to physically interact with the PPXY motif of various transcription factors via such domains and it has been postulated that one of its mechanisms of action is to impede nuclear translocation, thus regulating their transcriptional activity [5,49]. In this study, we propose that *via* the same mechanism WWOX acts as an inhibitor of TGFβ signaling by binding to SMAD3 and modulating nuclear translocation of this transcription factor, thus reducing promoter occupation and transcriptional activation. In the absence of WWOX, a condition that emulates advanced breast cancer, SMAD3 can enter the nucleus uninhibited. Promoter specificity and activation of pro-metastatic genes such as *ANGPTL4*, *PTHLH* and *SERPINE1*, depends on SMAD3 interaction with specific transcriptional co-activators such as RUNX2. RUNX2 is a SMAD3 coactivator that has been shown to induce EMT [50] and pro-metastatic genes such as *ANGPTL4*[33] in a TGFβ-dependent manner. Interestingly, it has been previously demonstrated that WWOX also binds to RUNX2 (Figure 5A) and modulates its transcriptional activity [32]. The ability of WWOX to affect the transcriptional activity of not only SMAD3 but also of a key transcriptional cofactor such as RUNX2 suggests that the presence or absence of WWOX could be critical for modulating TGFβ signaling and, more importantly, for the activation or repression of specific transcriptional targets known to be associated with tumor progression. Interestingly, our breast cancer gene expression meta-analysis indicates an inverse correlation between *WWOX* and *ANGPTL4.* Furthermore, tumors with the *WWOX*^*lo*^*/ANGPTL4*^*hi*^ signature correlate with breast cancer subtypes characterized by poor prognosis. Thus, the *WWOX*^*lo*^*/ANGPTL4*^*hi*^ breast cancer subset could represent good candidates for exploring anti-TGFβ therapeutic approaches.

## Conclusions

Loss of WWOX expression leads to significant upmodulation of SMAD3 transcriptional activity leading to overexpression of multiple gene targets associated with breast cancer progression. WWOX directly binds SMAD3 via WW domain 1 and inhibits its transcriptional activity by sequestering this transcription factor in the cytoplasmic compartment.

In summary, we hypothesize that the progressive loss of WWOX expression in advanced breast cancer contributes to deregulating the TGFβ pathway and, more importantly, may explain some of the pro-metastatic effects resulting from TGFβ/SMAD3 hyperactive signaling in advanced breast cancer.

## Abbreviations

WWOX: WW-domain containing oxidoreductase; ANGPTL4: Angiopoietin-like 4; FST: Follistatin; PTHLH: Parathyroid hormone-like hormone; SERPINE1: Serpin peptidase inhibitor, clade E, member 1; TGFβ: Transforming growth factor β; DMEM: Dulbecco’s modified Eagle’s medium; IMEM: Minimum Essential Medium; MEBM: Mammary epithelial basal medium; MOI: Multiplicity of infection.

## Competing interests

The authors declare that they have no competing interests.

## Authors’ contributions

CMA and BWF contributed the conception of the project and the design of all experiments. Experiments were conducted by BWF, XG, MJZ and JL. CRJ contributed confocal microscopy expertise. MCA carried out all bioinformatic analyses. BWF, MCA and CMA wrote the main body of the manuscript. All authors read and gave their final approval for the manuscript.

## Pre-publication history

The pre-publication history for this paper can be accessed here:

http://www.biomedcentral.com/1471-2407/13/593/prepub

## Supplementary Material

Additional file 1 **WWOX silencing in MCF10 cells results in decreased attachment to extracellular matrix substrates.** Attachment of MCF10 Scr control or shWWOX cells to laminin, collagen or fibronectin matrices. Click here for file

Additional file 2 **Microarray gene expression data and ChIP Enrichment Analysis data from WWOX-silenced MCF10 cells.** Genes up- or down-regulated in WWOX-silenced cells and the ChEA data of predicted transcription factors affected by WWOX silencing. Click here for file

## References

[B1] Bednarek AK Laflin KJ Daniel RL Liao Q Hawkins KA Aldaz CM WWOX, a novel WW domain-containing protein mapping to human chromosome 16q23.3-24.1, a region frequently affected in breast cancer Cancer Res 2000 60 8 2140 2145 10786676

[B2] Ried K Finnis M Hobson L Mangelsdorf M Dayan S Nancarrow JK Woollatt E Kremmidiotis G Gardner A Venter D Common chromosomal fragile site FRA16D sequence: identification of the FOR gene spanning FRA16D and homozygous deletions and translocation breakpoints in cancer cells Hum Mol Genet 2000 9 11 1651 1663 10.1093/hmg/9.11.1651 10861292

[B3] Bignell GR Greenman CD Davies H Butler AP Edkins S Andrews JM Buck G Chen L Beare D Latimer C Signatures of mutation and selection in the cancer genome Nature 2010 463 7283 893 898 10.1038/nature08768 20164919 PMC3145113

[B4] Ramos D Aldaz CM WWOX, a chromosomal fragile site gene and its role in cancer Adv Exp Med Biol 2006 587 149 159 10.1007/978-1-4020-5133-3_14 17163164

[B5] Aqeilan RI Croce CM WWOX in biological control and tumorigenesis J Cell Physiol 2007 212 2 307 310 10.1002/jcp.21099 17458891

[B6] Paige AJ Taylor KJ Taylor C Hillier SG Farrington S Scott D Porteous DJ Smyth JF Gabra H Watson JE WWOX: a candidate tumor suppressor gene involved in multiple tumor types Proc Natl Acad Sci U S A 2001 98 20 11417 11422 10.1073/pnas.191175898 11572989 PMC58744

[B7] Bergsagel PL Kuehl WM Chromosome translocations in multiple myeloma Oncogene 2001 20 40 5611 5622 10.1038/sj.onc.1204641 11607813

[B8] Nunez MI Ludes-Meyers J Abba MC Kil H Abbey NW Page RE Sahin A Klein-Szanto AJ Aldaz CM Frequent loss of WWOX expression in breast cancer: correlation with estrogen receptor status Breast Cancer Res Treat 2005 89 2 99 105 10.1007/s10549-004-1474-x 15692750 PMC4145848

[B9] Guler G Uner A Guler N Han SY Iliopoulos D Hauck WW McCue P Huebner K The fragile genes FHIT and WWOX are inactivated coordinately in invasive breast carcinoma Cancer 2004 100 8 1605 1614 10.1002/cncr.20137 15073846

[B10] Bednarek AK Keck-Waggoner CL Daniel RL Laflin KJ Bergsagel PL Kiguchi K Brenner AJ Aldaz CM WWOX, the FRA16D gene, behaves as a suppressor of tumor growth Cancer Res 2001 61 22 8068 8073 11719429

[B11] Qin HR Iliopoulos D Semba S Fabbri M Druck T Volinia S Croce CM Morrison CD Klein RD Huebner K A role for the WWOX gene in prostate cancer Cancer Res 2006 66 13 6477 6481 10.1158/0008-5472.CAN-06-0956 16818616

[B12] Nakayama S Semba S Maeda N Aqeilan RI Huebner K Yokozaki H Role of the WWOX gene, encompassing fragile region FRA16D, in suppression of pancreatic carcinoma cells Cancer Sci 2008 99 7 1370 1376 10.1111/j.1349-7006.2008.00841.x 18460020 PMC11159152

[B13] Aqeilan RI Trapasso F Hussain S Costinean S Marshall D Pekarsky Y Hagan JP Zanesi N Kaou M Stein GS Targeted deletion of Wwox reveals a tumor suppressor function Proc Natl Acad Sci U S A 2007 104 10 3949 3954 10.1073/pnas.0609783104 17360458 PMC1820689

[B14] Ludes-Meyers JH Kil H Parker-Thornburg J Kusewitt DF Bedford MT Aldaz CM Generation and characterization of mice carrying a conditional allele of the Wwox tumor suppressor gene PLoS One 2009 4 11 e7775 10.1371/journal.pone.0007775 19936220 PMC2777388

[B15] Ferguson BW Gao X Kil H Lee J Benavides F Abba MC Aldaz CM Conditional Wwox deletion in mouse mammary gland by means of two Cre recombinase approaches PLoS One 2012 7 5 e36618 10.1371/journal.pone.0036618 22574198 PMC3344920

[B16] Labbe E Silvestri C Hoodless PA Wrana JL Attisano L Smad2 and Smad3 positively and negatively regulate TGF beta-dependent transcription through the forkhead DNA-binding protein FAST2 Mol Cell 1998 2 1 109 120 10.1016/S1097-2765(00)80119-7 9702197

[B17] Wrana JL Attisano L Carcamo J Zentella A Doody J Laiho M Wang XF Massague J TGF beta signals through a heteromeric protein kinase receptor complex Cell 1992 71 6 1003 1014 10.1016/0092-8674(92)90395-S 1333888

[B18] Smyth GK limma: Linear Models for Microarray Data Bioinformatics and Computational Biology Solutions Using R and Bioconductor 2005 New York: Springer Verlag 397 420

[B19] Breitling R Armengaud P Amtmann A Herzyk P Rank products: a simple, yet powerful, new method to detect differentially regulated genes in replicated microarray experiments FEBS Lett 2004 573 1–3 83 92 15327980 10.1016/j.febslet.2004.07.055

[B20] Saeed AI Sharov V White J Li J Liang W Bhagabati N Braisted J Klapa M Currier T Thiagarajan M TM4: a free, open-source system for microarray data management and analysis Biotechniques 2003 34 2 374 378 12613259 10.2144/03342mt01

[B21] Smid M Dorssers LC Jenster G Venn Mapping: clustering of heterologous microarray data based on the number of co-occurring differentially expressed genes Bioinformatics 2003 19 16 2065 2071 10.1093/bioinformatics/btg282 14594711

[B22] Ingenuity Pathways Analysis http://www.ingenuity.com

[B23] Enrichr Online Resource http://amp.pharm.mssm.edu/Enrichr

[B24] Lachmann A Xu H Krishnan J Berger SI Mazloom AR Ma’ayan A ChEA: transcription factor regulation inferred from integrating genome-wide ChIP-X experiments Bioinformatics 2010 26 19 2438 2444 10.1093/bioinformatics/btq466 20709693 PMC2944209

[B25] Ludes-Meyers JH Kil H Bednarek AK Drake J Bedford MT Aldaz CM WWOX binds the specific proline-rich ligand PPXY: identification of candidate interacting proteins Oncogene 2004 23 29 5049 5055 10.1038/sj.onc.1207680 15064722 PMC4143251

[B26] Naruhn S Meissner W Adhikary T Kaddatz K Klein T Watzer B Muller-Brusselbach S Muller R 15-hydroxyeicosatetraenoic acid is a preferential peroxisome proliferator-activated receptor beta/delta agonist Mol Pharmacol 2010 77 2 171 184 10.1124/mol.109.060541 19903832

[B27] Kaddatz K Adhikary T Finkernagel F Meissner W Muller-Brusselbach S Muller R Transcriptional profiling identifies functional interactions of TGF beta and PPAR beta/delta signaling: synergistic induction of ANGPTL4 transcription J Biol Chem 2010 285 38 29469 29479 10.1074/jbc.M110.142018 20595396 PMC2937979

[B28] InSilico DB Genomic Datasets Hub http://insilico.ulb.ac.be/

[B29] Gaussian Mixture Modeling http://www.astro.lsa.umich.edu/~ognedin/gmm

[B30] Sudol M Hunter T NeW wrinkles for an old domain Cell 2000 103 7 1001 1004 10.1016/S0092-8674(00)00203-8 11163176

[B31] Selvamurugan N Kwok S Partridge NC Smad3 interacts with JunB and Cbfa1/Runx2 for transforming growth factor-beta1-stimulated collagenase-3 expression in human breast cancer cells J Biol Chem 2004 279 26 27764 27773 10.1074/jbc.M312870200 15084595

[B32] Aqeilan RI Hassan MQ de Bruin A Hagan JP Volinia S Palumbo T Hussain S Lee SH Gaur T Stein GS The WWOX tumor suppressor is essential for postnatal survival and normal bone metabolism J Biol Chem 2008 283 31 21629 21639 10.1074/jbc.M800855200 18487609 PMC2490770

[B33] Padua D Zhang XH Wang Q Nadal C Gerald WL Gomis RR Massague J TGFbeta primes breast tumors for lung metastasis seeding through angiopoietin-like 4 Cell 2008 133 1 66 77 10.1016/j.cell.2008.01.046 18394990 PMC2390892

[B34] Zhu P Tan MJ Huang RL Tan CK Chong HC Pal M Lam CR Boukamp P Pan JY Tan SH Angiopoietin-like 4 protein elevates the prosurvival intracellular O2(-): H2O2 ratio and confers anoikis resistance to tumors Cancer Cell 2011 19 3 401 415 10.1016/j.ccr.2011.01.018 21397862

[B35] Aqeilan RI Donati V Gaudio E Nicoloso MS Sundvall M Korhonen A Lundin J Isola J Sudol M Joensuu H Association of Wwox with ErbB4 in breast cancer Cancer Res 2007 67 19 9330 9336 10.1158/0008-5472.CAN-07-2147 17909041

[B36] Guler G Huebner K Himmetoglu C Jimenez RE Costinean S Volinia S Pilarski RT Hayran M Shapiro CL Fragile histidine triad protein, WW domain-containing oxidoreductase protein Wwox, and activator protein 2gamma expression levels correlate with basal phenotype in breast cancer Cancer 2009 115 4 899 908 10.1002/cncr.24103 19130459 PMC2640223

[B37] Bos PD Zhang XH Nadal C Shu W Gomis RR Nguyen DX Minn AJ van de Vijver MJ Gerald WL Foekens JA Genes that mediate breast cancer metastasis to the brain Nature 2009 459 7249 1005 1009 10.1038/nature08021 19421193 PMC2698953

[B38] Janicke F Schmitt M Pache L Ulm K Harbeck N Hofler H Graeff H Urokinase (uPA) and its inhibitor PAI-1 are strong and independent prognostic factors in node-negative breast cancer Breast Cancer Res Treat 1993 24 3 195 208 10.1007/BF01833260 8435475

[B39] Silberstein GB Daniel CW Reversible inhibition of mammary gland growth by transforming growth factor-beta Science 1987 237 4812 291 293 10.1126/science.3474783 3474783

[B40] Barcellos-Hoff MH Akhurst RJ Transforming growth factor-beta in breast cancer: too much, too late Breast Cancer Res 2009 11 1 202 10.1186/bcr2224 19291273 PMC2687712

[B41] Moses H Barcellos-Hoff MH TGF-beta biology in mammary development and breast cancer Cold Spring Harb Perspect Biol 2011 3 1 a003277 20810549 10.1101/cshperspect.a003277 PMC3003461

[B42] Padua D Massague J Roles of TGFbeta in metastasis Cell Res 2009 19 1 89 102 10.1038/cr.2008.316 19050696

[B43] Massague J TGFbeta signalling in context Nat Rev Mol Cell Biol 2012 13 10 616 630 10.1038/nrm3434 22992590 PMC4027049

[B44] Chen CR Kang Y Siegel PM Massague J E2F4/5 and p107 as Smad cofactors linking the TGFbeta receptor to c-myc repression Cell 2002 110 1 19 32 10.1016/S0092-8674(02)00801-2 12150994

[B45] Feng XH Liang YY Liang M Zhai W Lin X Direct interaction of c-Myc with Smad2 and Smad3 to inhibit TGF-beta-mediated induction of the CDK inhibitor p15(Ink4B) Mol Cell 2002 9 1 133 143 10.1016/S1097-2765(01)00430-0 11804592

[B46] Pardali K Kurisaki A Moren A ten Dijke P Kardassis D Moustakas A Role of Smad proteins and transcription factor Sp1 in p21(Waf1/Cip1) regulation by transforming growth factor-beta J Biol Chem 2000 275 38 29244 29256 10.1074/jbc.M909467199 10878024

[B47] Aragon E Goerner N Zaromytidou AI Xi Q Escobedo A Massague J Macias MJ A Smad action turnover switch operated by WW domain readers of a phosphoserine code Genes Dev 2011 25 12 1275 1288 10.1101/gad.2060811 21685363 PMC3127429

[B48] Sudol M WW domains in the heart of Smad regulation Structure 2012 20 10 1619 1620 10.1016/j.str.2012.09.007 23063008

[B49] Salah Z Aqeilan R Huebner K WWOX gene and gene product: tumor suppression through specific protein interactions Future Oncol 2010 6 2 249 259 10.2217/fon.09.152 20146584 PMC2832309

[B50] Chimge NO Baniwal SK Little GH Chen YB Kahn M Tripathy D Borok Z Frenkel B Regulation of breast cancer metastasis by Runx2 and estrogen signaling: the role of SNAI2 Breast Cancer Res 2011 13 6 R127 10.1186/bcr3073 22151997 PMC3326569

