# Supplementary material for: The cancer gene WWOX behaves as an inhibitor of SMAD3 transcriptional activity via direct binding
Source: BMC Cancer. 2013 Dec 11;13:593. doi: 10.1186/1471-2407-13-593 (PMC3871008; doi:10.1186/1471-2407-13-593)
Supplement: Additional file 1 — WWOX silencing in MCF10 cells results in decreased attachment to extracellular matrix substrates. Attachment of MCF10 Scr control or shWWOX cells to laminin, collagen or fibronectin matrices. [file 1471-2407-13-593-S1.docx]

**
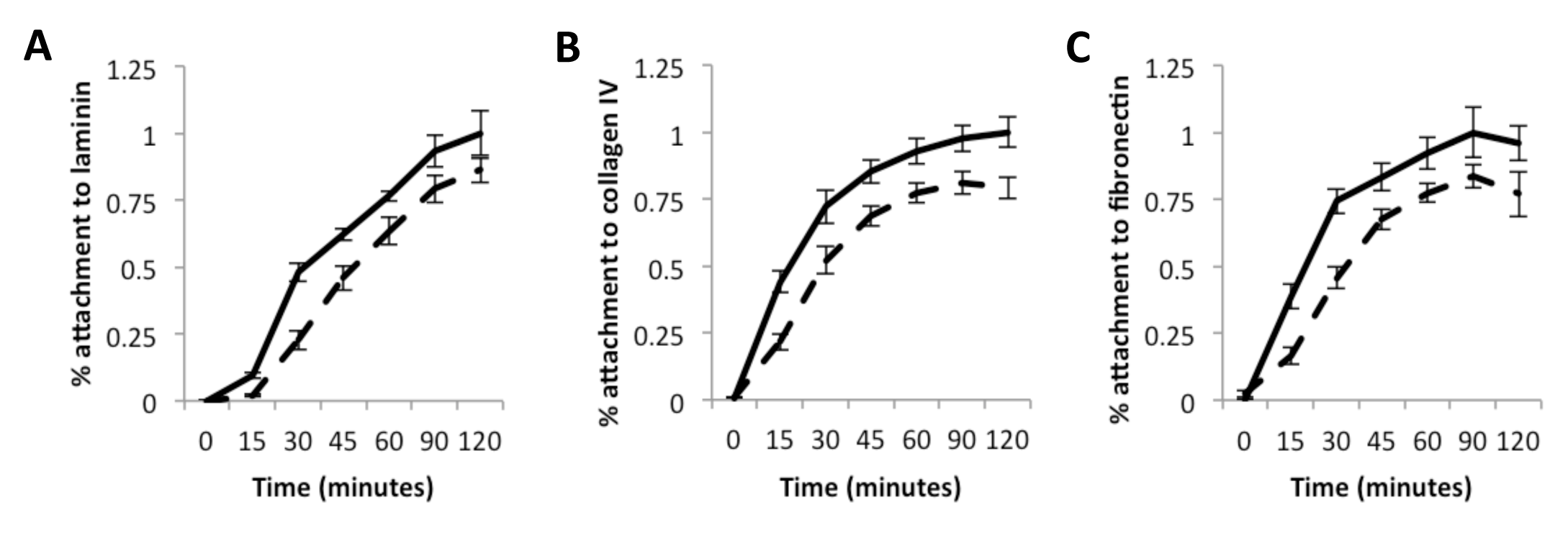
**

**Additional File 1. WWOX silencing in MCF10 cells results in decreased attachment to extracellular matrix substrates.**  Effect of *WWOX* knockdown on attachment of MCF10 cells to laminin **(A)**, collagen IV **(B)** and fibronectin **(C).** Cell adhesion measured by the SRB assay. Solid line, Scr subline; dashed line, sh*WWOX* subline; data points represent the average of two independent experiments done in quadruplicate ± SEM.
